# The Importance of Diagnosing Concomitant Delirium and Catatonia: A Case Report

**DOI:** 10.7759/cureus.21662

**Published:** 2022-01-27

**Authors:** Aquila Lesko, Naciye Kalafat, Khadijah Enoh, Warren K Teltser

**Affiliations:** 1 Psychiatry, Medical College of Wisconsin Green Bay, Green Bay, USA; 2 Psychiatry, Bellin Psychiatric Center, Green Bay, USA; 3 Clinical Sciences, Medical College of Wisconsin Green Bay, Green Bay, USA; 4 Clinical Sciences, Medical University of Lublin, Lublin, POL

**Keywords:** benzodiazepine use in delirium, antipsychotic use in catatonia, co-occurring delirium and catatonia, delirium, catatonia

## Abstract

Catatonia syndrome is characterized by motor, behavioral and affective abnormalities in association with psychiatric and medical illnesses and delirium syndrome is defined as acute brain dysfunction caused by an underlying medical condition or toxic exposure. The Diagnostic and Statistical Manual of Mental Disorders, 5th Edition (DSM-5) contains a caveat that limits diagnosing catatonia in patients during delirium. However, the literature has shown that up to 31% of patients have co-occurring catatonia and delirium when using the Bush Francis Catatonia Rating Scale and 12.7% of patients with delirium meet DSM-5 criteria for catatonia. The authors present a case of a patient with concomitant delirium and catatonia. Diagnosing catatonia in this patient, even in the setting of delirium, was necessary for appropriate treatment and clinical improvement. Typical treatment for patients with delirium, antipsychotic medication, contributes to the worsening of catatonia while first-line treatment for catatonia, benzodiazepines, has been shown to exacerbate delirium. Delayed recognition of the patient’s catatonia resulted in inadequate treatment that worsened her catatonic symptoms and prolonged hospitalization. The potential contraindications to treatment interventions call for an appropriate diagnosis of catatonia when co-occurring with delirium despite the DSM-5 limitation. The World Health Organization (WHO) ICD-11 code for catatonia allows for less exclusivity in assessing for clinical catatonia in that the limitations to diagnosis only include harmful effects of drugs, medicaments or biological substance, not elsewhere classified - a more collaborative definition for catatonia criteria in the DSM-5 and the ICD-11 codes can provide a way forward with more flexibility in symptom interpretation and treatment.

## Introduction

Catatonia is a syndrome characterized by prominent motor, behavioral and affective abnormalities that occur in both psychiatric and medical illnesses [1]. The estimated prevalence of catatonia amongst psychiatric inpatient patients is about 10%, with 30% suffering from schizophrenia, 43% from bipolar, and up to 25% from a general medical or neurological condition [2]. Other conditions linked to catatonia include obsessive-compulsive disorder, post-traumatic stress disorder, and withdrawal from alcohol or benzodiazepines. Catatonia has an excellent prognosis if recognized and treated appropriately, but it has the potential to be lethal when accompanied by fever and autonomic disturbances [2]. Termed “malignant catatonia,” this dangerous form of the syndrome presents as stuporous exhaustion, respiratory failure, collapse, coma, and possibly death. Therefore, early recognition and prompt treatment of catatonia are extremely important.

Interestingly enough, the Diagnostic and Statistical Manual of Mental Disorders, 5th Edition (DSM-5) specifically states “A separate diagnosis of catatonic disorder due to another medical condition is not given if catatonia occurs exclusively during the course of a delirium” [3]. However, there is increased recognition of catatonic signs in delirium patients [4-6] with research suggesting that up to one-third of critically ill patients experience co-occurring delirium and catatonia [4,6]. Delirium is also a syndrome, but of acute brain dysfunction caused by an underlying medical condition or toxic exposure [3]. It is characterized by deficits in awareness, attention, and cognition and is the most common psychiatric syndrome observed in medically hospitalized patients. It is more easily identified as it occurs in one out of five medical inpatients, two out of five intensive care unit patients, and up to four out of five mechanically ventilated patients with critical illness [5]. Catatonia, on the other hand, is often a missed diagnosis and results in prolonged altered mental status (AMS) in patients diagnosed with delirium.

Evidence is mounting that delirium and catatonia can coexist despite the DSM-5 exclusion criteria. Using the Bush Francis Catatonia Rating Scale (BFCSR) and the DSM-5 criteria for catatonia, Grover and colleagues determined that 30.2% and 12.7%, respectively, of 205 delirium subjects qualified for a catatonia syndrome diagnosis. The study also found that 40% of those subjects had at least two of the first 14 items on the BFCSR, a sensitive indicator of catatonia [4]. Wilson et al. found similar figures using the BFCRS mapped to DSM-5 criterion: of 136 critically ill patients on mechanical ventilation and/or vasopressors, 31% had co-occurring catatonia and delirium [6]. A study by Jaimes-Albornoz and Serra-Mestres screened 112 patients over 65 years old with the BFCRS and found that 10 patients met research diagnostic criteria for catatonia. Of these 10, three (30%) had concomitant delirium [7]. In 2009, Francis and Lopez-Canino applied three methods to identify cases of delirium with catatonic features: using BFCSR on published case studies of delirium, reassessment of their earlier 20 published cases of drug-induced catatonia, and including their three most recent cases of delirium that met research criteria of catatonia with the BFCSR. Using these methods, the study identified 16 patients who met the criteria for concomitant delirium and catatonia [8]. In particular relevance to the case presented below, two studies found that 30%-50% of elderly patients in the acute medical setting diagnosed with catatonia had concomitant delirium [7,9].

Below is a case presentation of a patient who also met diagnostic criteria for concomitant delirium and catatonia. Additionally, the case will outline the importance of acknowledging a diagnosis of catatonia even though delirium is present, regardless of the DSM-5 criteria, because of the treatment implications.

## Case presentation

A 66-year-old female with a past psychiatric history of bipolar disorder type 1 and medical history of diabetes type II and recurrent urinary tract infection (UTI) presented to the emergency department with auditory hallucinations, an extremely flat affect, and suicidal ideation presumably due to delirium syndrome secondary to a UTI. The patient had been admitted to the medical floor and after ceftriaxone and fluid treatment she experienced resolution of symptoms. After antibiotic treatment, her urine cultures and sensitivity returned showing normal flora and the patient was transferred to the inpatient psychiatric unit as the patient felt her mood symptoms were inadequately managed since her lithium treatment was discontinued. The patient’s bipolar disorder had been well managed on lithium for almost thirty years but had caused acute kidney injury two months previously, and she was experiencing periodic issues with her mood symptoms since.

The patient's home medication, alprazolam 1mg nightly, metformin 24 hour 500mg daily and risperidone 1mg BID, were restarted on admission. Of note, the patient's A1c was 7.9, indicating poor glycemic control. On day 1, the patient was logical, coherent, alert, and oriented x2 with some confusion pertaining to time. Her episodic mood symptoms were considered to be related to the discontinuation of lithium so she was started on divalproex sodium 500mg nightly. During the evening of day 1, the patient was noted to be talking to herself and would make nonsensical statements about time and death to staff members. On day 2, the patient presented with confusion pertaining to time and preoccupations with the thought that she may die. She reported her thoughts of dying were lessened and a dose of divalproex sodium 250mg was started in the morning. The patient's nightly risperidone was increased to 1.5mg for worsening mental status at nighttime. Because the patient had recurrent UTIs but was not started on prophylactic antibiotics, a repeat urinalysis (UA) was collected and prophylactic trimethoprim/sulfamethoxazole 800-160mg BID was initiated while awaiting results. During the evening of day 2, the patient was noted to stare blankly, walk the halls without her pants on, and be intrusive with peers. Per staff, the patient was in and out of lucidity and made more nonsensical statements pertaining to death. She slept a total of two hours. 

On day 3, the patient was noted to still be intrusive of peers and needed frequent redirecting due to confusion. Her UA was positive for cloudy urine, RBCs, leukocyte esterase, packed WBCs, bacteria +2, and yeast +4. Internal medicine was consulted upon receiving the results and she was treated with oral fosfomycin 3mg and fluconazole 150mg every 72 hours for two doses. During pre-rounds on day 3, the patient was extremely confused, had delusions, experienced auditory hallucinations, and exhibited stereotypy. She had a masked face with an intense stare and an extremely flat affect. However, upon returning hours later, the patient’s confusion had drastically subsided. She was lucid and coherent, denied her previously delusional thoughts, and demonstrated insight by recognizing that she had been in an altered state of consciousness.

One day after receiving treatment for her combined bacterial and fungal UTI, the patient appeared to be at baseline. Her masked face had dissolved and her affect was bright. She had slept well the night previously and was experiencing much less confusion, being oriented to person, place, and time. She was agreeable to simplifying her medication regime by combining her divalproex sodium doses to 750mg daily at bedtime. However, on the night of day 4, the nursing staff noted the patient continuously knocked on the nurse’s station without needing anything. She was staring blankly and was not redirectable to her room. She was noted to interrupt staff during a new admit to the unit because she believed the arriving peer was her husband. The patient only slept one hour that night.

On day 5, the patient was observed to be speaking to herself. She had an extremely flat affect once again, demonstrating a pronounced delay in answering questions while staring intensely. She answered many questions with nonsensical statements and voiced persecutory delusions about death and being dead. During the interview, the patient exhibited stereotypy by resting her hands on her knees and flapping both hands at the wrist repeatedly. An additional dose of risperidone 0.5mg at 1500 was scheduled to address the cyclic nature of her confusion in the afternoon. The patient was noted to sleep better overnight and the patient was coherent and logical the next morning. She had a bright affect and demonstrated much less confusion. She was able to outline how her night went and articulate that she was not feeling well the day previously. Approximately two hours after this initial interview, however, the patient was noticed to have a slow, shuffling gait with decreased arm swinging and masked faces. She was confused and entered several different patients’ rooms that were not hers. Cogwheel rigidity test revealed increased rigidity. The patient was noted to repeat phrases she heard on the unit and mimicked a staff member’s motion of putting hands on her hips.

Due to the patient’s apparent echopraxia and echolalia, the Bush-Francis Catatonia Scale was applied. The patient scored a 17 for immobility, mutism, staring, posturing, echopraxia/echolalia, stereotypy, verbigeration, rigidity, negativism, impulsivity (for removing clothing and walking down the halls naked), and perseveration. Collateral was collected from the patient's husband that further confirmed a waxing and waning catatonic state for almost two months as he endorsed the patient experienced episodic staring, immobility, echolalia, negativism, verbigeration, perseveration, and combativeness. The patient was given a lorazepam 1mg PO challenge test for catatonia. One-hour post-test, the patient was not noted to exhibit any significant improvement. After two hours, the patient’s affect was bright and her thought content was logical and coherent. Her gait was noted to be much improved and her confusion had subsided. She no longer made nonsensical statements or exhibited echolalia/echopraxia.

The patient was started on 1mg lorazepam TID for catatonic behavior, her risperidone 1.5mg daily at bedtime was decreased to 1.0mg as antipsychotics can worsen catatonia, and her scheduled and PRN alprazolam doses were discontinued to avoid benzodiazepine polypharmacy on day 6. That night, the patient was noted to resist sleep, exhibit disorientation to time, pace in the hallways and stare at the television while it was off. Her last dose of lorazepam was given at 1800 that day. The morning of day 7, the patient had a bright affect and was able to acknowledge that her confused states fluctuated, often getting worse at night and clearing in the morning. Her thoughts were logical and coherent and her gait was normal. Her lorazepam was staggered for more appropriate coverage throughout the day: 1mg BID at 0800 and 1800 and 2mg BID at 1300 and 2200. Repeat UA at this time indicated that the patient’s yeast and bacterial urinary tract infection had resolved. Her divalproex sodium level was drawn and found to be at the therapeutic level of 51.6mcg/mL 1800 hours after the patient’s last dose.

Due to sedation and slurring of words on day 8, the on-call nurse practitioner discontinued all lorazepam doses except for the 1mg morning dose on day 9. This was immediately corrected by increasing the lorazepam dose to 1.0mg TID on day 10. The patient was noted to exhibit mild, intermittent episodes of confusion on day 9 but slept overnight and did not exhibit overtly catatonic behavior. On day 10, she had a bright affect with a logical and coherent thought process. Her gait was normal and the patient's husband reported that her behavioral presentation was at baseline. Overnight, the patient slept well and exhibited no episodes of confusion or catatonic behavior. On day 11, the patient’s bright affect still remained. She had been noted to maintain coherency and orientation for over 24 hours. On day 11, the patient was discharged on lorazepam 1mg TID and divalproex sodium 750mg daily at bedtime.

## Discussion

In summary, due to the patient’s psychiatric history of bipolar disorder and her presenting mood symptoms, the patient required mood stabilization resulting in divalproex sodium being administered. When residual delirium was noted via sundowning, her risperidone dose was increased. As the patient’s AMS continued to worsen, a UTI was added to the differential and the patient was treated for it with the expectation that the AMS symptoms would resolve. Unfortunately, her symptoms only temporarily improved only to descend back into the previous pattern of waxing and waning AMS. However, this time, she exhibited more pronounced symptoms of masked facies, stereotypes, and additional symptoms such as a slow, shuffling gait, increased rigidity, repetition of phrases, and mimicry of staff. At this point, catatonia was diagnosed and treated with lorazepam while lowering her dose of antipsychotics to preclude the worsening of her catatonic state. Ultimately, the administration of benzodiazepines resulted in the full resolution of her delirium syndrome.

The differential diagnoses considered were risperidone-induced parkinsonism and Lewy body dementia. Risperidone-induced parkinsonism was explored due to the time course of her dose increase of risperidone and the exhibited shuffling gait and muscular rigidity. The second differential was Lewy body dementia because of her visual hallucinations and decline in neurocognitive ability with antipsychotic use. However, catatonia remains the primary differential because of her improvement with lorazepam. Furthermore, a review of the patient’s chart revealed no previous diagnosis of dementia, and collateral information from her spouse showed no observation of prior cognitive impairment. Lastly, seizures were considered since she was noted to have a staring spell accompanied by immobility; however, electroencephalography was not done during this hospital stay to confirm or deny its occurrence.

While the primary treatment intervention for a patient with delirium is individualized to the clinical presentation and includes assessment and modification of environmental factors, it remains that the medication of choice for delirium is short-term low-dose antipsychotic therapy. A 2016 meta-analysis of antipsychotic treatment in patients with delirium found that antipsychotics were superior to placebo or usual care in terms of response rate (RR = 0.22, NNT = 2), delirium severity scales scores (SMD = -1.27), GGI-S scores (SMD = -1.57), and time to response (SMD = -1.22) [10]. In a multi-center observational study, risperidone specifically was effective (effectiveness measured using the Trzepacz Delirium Rating Scale, the Positive Subscale of the PANSS, the Mini-Mental Status Examination, and the Clinical Global Impressions Scale) in 90.6% of 64 patients who met the criteria for delirium and with fewer side effects than other medications such as haloperidol [11].

Treatment of delirium with benzodiazepines is contraindicated except for in two settings sedative drug and alcohol withdrawal and when antipsychotic medications are contraindicated. In a systematic review of benzodiazepines used in cases of delirium, one study showed less effectiveness in benzodiazepines compared to antipsychotic treatment [12]. In another study, lorazepam was associated with increased delirium compared to dexmedetomidine, with less days alive without delirium or coma (median 3.0 vs 7.0; p = 0.01), higher prevalence of coma (92% vs 63%; p = 0.001), lower percentage of days spent within 1 Richmond Agitation-Sedation Scale point of the sedation goal (median % of days, 67% vs 80%; p = 0.04) and a higher 28-day mortality (27% vs 17%; p = 0.18) [13]. Benzodiazepines, along with other sedative medications, are even known to be a precipitating factor of delirium [14]. While the list of precipitating factors is long, benzodiazepines were found to have the most consistent and strong association with an increased burden of delirium in this systematic review.

The recommended first-line treatment for catatonia is based on the subtype of catatonia and clinical urgency (with much also depending on the availability of the treatment options), requiring either benzodiazepine therapy or electroconvulsive therapy (ECT) [15]. Yet while benzodiazepines have clear indications for catatonia, the use of antipsychotics as treatment is questionable. A systematic review of catatonia notes that historical literature demonstrates antipsychotics causing worsening in symptomatology, clouding the clinical presentation with parkinsonism, causing neuroleptic-induced catatonia, and inducing neuroleptic-malignant syndrome in catatonic patients [16]. Recommendations typically include discontinuation of neuroleptic medication if feasible, but if psychiatric comorbidities do not allow discontinuation then it is recommended to avoid higher potency antipsychotics and monitor closely for adverse effects.

It is important to highlight that both the criteria to identify and treat delirium and catatonia strongly keep them as separate entities. The DSM-5 suggests that treatment teams should avoid diagnosing catatonia when a patient shows the symptoms exclusively during delirium, and catatonia treatment guidelines show that antipsychotics, a treatment for delirium, can exacerbate catatonic symptoms. This presents a treatment conundrum when a patient presents with both. How does one parse out one diagnosis from another or treat two contraindicative diagnoses in one body? In this patient’s case, the delirium took precedence, and this ultimately prolonged her catatonic features, allowing them to worsen over her 11-day inpatient stay.

The WHO ICD-11 code for catatonia allows for less exclusivity in assessing for clinical catatonia in that the limitations to diagnosis only include harmful effects of drugs, medicaments or biological substances, not elsewhere classified [17]. The hope for a more collaborative definition for catatonia criteria in the DSM-5 and the WHO ICD-11 codes can provide a way forward with more flexibility in symptom interpretation and treatment precedence for patients such as the one presented here.

## Conclusions

If the DSM-5 recommendation is to keep these diagnoses mutually exclusive, many patients will end up misdiagnosed, underrated, and with excessive hospital stays. If catatonia syndrome cannot be diagnosed in the setting of delirium syndrome, it is plausible that antipsychotic medication will be used in the treatment course of patients with delirium syndrome, thus potentially worsening or masking underlying catatonia syndrome. It is also possible that, since it is common practice to avoid benzodiazepine use in patients presenting with delirium, the catatonia will go undiagnosed longer and the time to resolution will be prolonged. It would be clinically beneficial if the next DSM revision removed the caveat that catatonia cannot be diagnosed in the setting of delirium, since the two diagnoses may have contradictory treatments. Investigating how to treat nuanced presentations such as the one in this case, without losing the points that keep them as discrete diagnoses, is worthy of further research for medication development and guidelines. The WHO ICD-11 code for catatonia allows for less exclusivity in assessing for clinical catatonia in that the limitations to diagnosis only include harmful effects of drugs, medicaments, or biological substances, not elsewhere classified. The hope fordelirium, the catatonia will go undiagnosed longer and a more collaborative definition for catatonia criteria in the DSM-5 and the WHO ICD-11 code can provide a way forward with more flexibility in symptom interpretation and treatment precedence for patients such as the one presented here.
